# On the Formation and Dynamics of Micro Dew Droplets on Grass: the Role of Epicuticular Wax

**DOI:** 10.1002/smll.202502219

**Published:** 2025-08-21

**Authors:** Bashra Mahamed, Francis James Dent, Robert Simpson, Nicola Weston, Maria S Vorontsova, Fanny Nascimento Costa, Sepideh Khodaparast

**Affiliations:** ^1^ School of Physics and Astronomy University of Leeds Leeds LS2 9JT UK; ^2^ School of Mechanical Engineering University of Leeds Leeds LS2 9JT UK; ^3^ School of Chemical and Process Engineering University of Leeds Leeds LS2 9JT UK; ^4^ Nanoscale & Microscale Research Centre (nmRC) University of Nottingham University Park Nottingham NG7 2RD UK; ^5^ Royal Botanic Gardens, Kew London, Richmond TW9 3AE UK; ^6^ The Bragg Centre for Materials Research Sir William Henry Bragg Building University of Leeds Leeds LS2 9JT UK

**Keywords:** epicuticular wax, drop‐wise condensation, dew, grass, jumping droplets

## Abstract

Though ubiquitous in everyday life, the formation of dew on grass arises from a precise balance of environmental conditions and surface microstructure. While condensation requires sufficient atmospheric moisture availability and cooling below the dew point, the formation of stable, spherical droplets is dependent on specialized surface architectures that promote nucleation and resist total wetting. Here, a closer look at the formation, growth, and dynamics of microscale dew droplets on the surface of wheatgrass leaves, investigating the role of epicuticular wax, is provided. The wheatgrass leaf exhibits biphilic properties emerging from the hydrophilic lamina covered by hydrophobic wax microsculptures, therefore, dew formation and dynamics are largely governed by the arrangement and density of epicuticular wax. Drop‐wise condensation is observed, resulting in discrete, highly mobile dew droplets on the superhydrophobic adaxial side, while the abaxial surfaces, characterised by reduced wax coverage, yield significant flooding and film‐wise condensation. Frequent coalescence of multiple droplets of 5–20 µm diameter on the adaxial side results in self‐propelled departure events, creating free potential sites for new nucleation. This dynamic regime of jumping dew droplets may provide a source of fresh water to surfaces and organisms in the vicinity of low grasses, considering their quasi‐vertical orientation.

## Graphical abstract

Despite their familiar appearance, dew droplets on grass emerge from a finely tuned interplay between environmental factors and surface architecture. Microscale analysis uncovers a key role of epicuticular wax micromorphologies in promoting the nucleation and growth of spherical droplets on the biphilic leaves of wheatgrass.

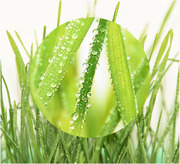

## 1 Introduction

Dew and related non‐rainfall inputs such as fog can provide 1–25% of the local water budget, especially in arid and semi‐arid environments, shown to sustain biodiversity, aid seedling establishment, and support the fauna dependent on moisture.^[^ ^1^, ^2^, ^3^ ^]^ While fog collection is limited to certain coastal and mountainous areas, dewfall is more common, as 99% of atmospheric moisture exists in vapour form.^[^ ^4^, ^5^, ^6^ ^]^ This is especially relevant in natural ecosystems where rainfall is scarce, providing a supplemental water source that supports plant and microbial activity, reduces early morning transpiration, and helps maintain soil moisture.^[^ ^7^, ^8^, ^9^ ^]^ While dew formation benefits ecosystems by buffering against water stress,^[^ ^10^ ^]^ persistent leaf wetness can also create conditions favorable for foliar pathogens, further reflecting the complex ecological role of dew.^[^ ^11^ ^]^

In nature, dew formation often relies on radiative cooling, where moisture from the air condenses on chilled surfaces.^[^ ^12^ ^]^ Interfacial condensation occurs as the surface temperature drops below that of the dew point, allowing water collection on surfaces such as plants, rocks, or engineered materials with high thermal emissivity.^[^ ^12^, ^13^, ^14^, ^15^ ^]^ This passive cooing strategy does not require external energy inputs and is largely dependent on environmental conditions.^[^ ^16^, ^17^ ^]^ Dew collection through radiative cooling is especially efficient across diverse species of plants inhabiting arid environments as a mechanism for collecting water from alternative resources,^[^ ^18^, ^19^, ^20^ ^]^ with identified examples native to a variety of arid and desert environments providing water sources for arthropods and self irrigation.^[^ ^21^, ^22^, ^23^, ^24^ ^]^ Studies on effective natural designs for dew collection in plants have provided a continuous source of inspiration for a variety of innovative engineered materials and systems designed for heat transfer and water harvesting applications in the past decades.^[^ ^18^, ^25^ ^]^ While previous investigations have focused primarily on the identification of dew collection mechanisms in plant species known for their specialised adaptations to survive in extremely hot and arid environments, this research aims to investigate dew formation in a common widespread family of monocotyledonous flowering plants known as grasses.

Grasses (family Poaceae) are among the most widespread and ecologically significant plant families on Earth, comprising around 12 000 species adapted to diverse terrestrial habitats, from arctic tundra to tropical savannas.^[^ ^26^, ^27^ ^]^ They dominate grasslands, savannas, steppes, and prairies, which together cover approximately 40% of the global land surface, and play a critical role in supporting biodiversity, carbon sequestration, and food security.^[^ ^28^, ^29^ ^]^ Grasses exhibit simple architectures with yet remarkable diversity in form, function, and ecological role, ranging from short turf grasses to tall bamboos.^[^ ^27^ ^]^ The multiscale plant architecture in grasses provide a potetially suitable design to perform as efficient natural dew collectors thanks to their significant interfacial area relative to the plant footprint, protection from high speed wind and effective and rapid radiative cooling enabled by the high emissivity and large surface to volume ratio.^[^ ^30^, ^31^ ^]^ The significance of dew contribution in plant health and population, alongside its contribution to the overall water budget has been identified and characterised in grasslands of varied climates with selected recent examples highlighted here. In low mountain ranges and alpine grasslands located in Austria and Germany, dew formation was found to significantly contribute to the water budget, generating 16% to 38% of the total monthly precipitation in dry and cold periods, respectively.^[^ ^32^ ^]^ The formation of dew occurred on ≈40% of the nights over five years of measurements in tropical semi‐arid grasslands of Mexico; dew water contributed to 5‐10% of annual precipitation, considerably balancing water stress during the dry months.^[^ ^2^ ^]^ Measurements over a Summer period in Hunskandak Sandlands, Inner Mongolia dominated by elmwoods and various species of grasses, namely *Agropyron criststum*, *Leymus chinensis*, *Cleistogenes squarrosa*, found overall larger amount of dew occurring over an average of 6 h per night in areas covered by grass, significantly influenced by the air temperature, relative humidity and plant coverage.^[^ ^33^ ^]^ Furthermore, regular dewfall was recorded on 54% of the summer and autumn nights in the Mongolia Plateau, resulting in significant water uptake in grasses ‐ relieving foliar water stress and supporting continuous growth of the plants in dry periods.^[^ ^15^ ^]^ Consistent dewfall was recorded on 78% of the nights during a 4‐year study in semi‐arid Mediterranean coastal ecosystems dominated by *Stipa tenacissima* grass, balancing ≈10–25% of the overall precipitation and providing 94% of the overall water balance during dry periods.^[^ ^34^ ^]^ When comparing the dew amount collected by various plants, grasses were significantly more efficient relative to sedges and forbes in a field study performed in alpine grasslands of the Tibetan Plateau, possibly due to the their larger overground biomass.^[^ ^35^ ^]^ Comparative studies between the taller *Leymus chinensis* (LC) and shorter *Cleistogenes squarros* (CS) grasses concluded that a consistently larger amount of dew was collected on CS both at plant scale and per unit organic matter, potentially explaining the succession from LC to the shorter CS under water stress. Although dew volumes are generally modest compared to rainfall, their contribution is often consistent and their cumulative ecological significance is substantial, underpinning the resilience, productivity, and biodiversity of grasslands.^[^ ^11^ ^]^ To date, while constant and significant ecological and biological impact of dew has been identified within grasslands, studies focused on the contributions of grass architecture, especially at smaller scales remain rare.

As dew formation relies on condensation nucleation and manipulation of small water droplets on the surface,^[^ ^36^ ^]^ the physical topography and interfacial energy of the surface across length scales play crucial roles in the efficiency of the designs.^[^ ^37^, ^38^, ^39^ ^]^ As a dominant group of organisms on the earth, plants occupy a large surface area on our planet; their leaves act as natural water regulators, collecting and controlling moisture under changing climatic circumstances.^[^ ^40^, ^41^ ^]^ Barthlott *et al*. estimated that the surface area covered by water repellent plant leaves, such as those found in grasses, is equal to ≈50% of the total surface of the earth.^[^ ^42^ ^]^ The external surface of the majority of plant leaves comprises a cuticle that is covered by a thin waxy layer, acting as a hydrophobic barrier.^[^ ^43^, ^44^ ^]^ This epicuticular layer is composed of crystalline waxes, typically mixtures of aliphatic hydrocarbons and their derivatives, such as primary and secondary alcohols, ketones, fatty acids, and aldehydes.^[^ ^45^ ^]^ While the significance of the micromorphology of epicuticular wax on the wetting behavior of the plant leaves has been extensively studied in relation to micro/macroscopic contact with water droplets,^[^ ^46^, ^47^, ^48^, ^49^, ^50^, ^51^, ^52^ ^]^ the link between the form and distribution of the epicuticular wax and the collection of microscopic dew droplets on the leaf is not yet fully understood due to the complex spatiotemporal dynamics of the process.^[^ ^53^, ^54^, ^55^ ^]^ Currently, studies on dew formation on plants and specially grasses are largely focused on macroscale field measurements, the modeling of foliar water uptake, and their ecological consequences at various environmental conditions.^[^ ^1^, ^8^, ^15^, ^56^, ^57^, ^58^, ^59^, ^60^, ^61^ ^]^ Previous works have highlighted the role of plant architecture or millimetric features on the surface of plants, such as ridges, hairs, spines and trichomes, in collecting fog droplet and dew in different species of plants.^[^ ^4^, ^22^, ^62^, ^63^, ^64^ ^]^ Quantitative analysis of larger millimetric dew and fog droplets has also been reported on grasses *Holcus lanatus* ^[^ ^58^ ^]^ and *Stipagrostis sabulicola*.^[^ ^22^ ^]^ In comparison, despite its important role in regulating the dew formation at early stages of nucleation and growth, the significance of the plant wax microstructure is less studied.^[^ ^65^ ^]^

**Figure 1:**
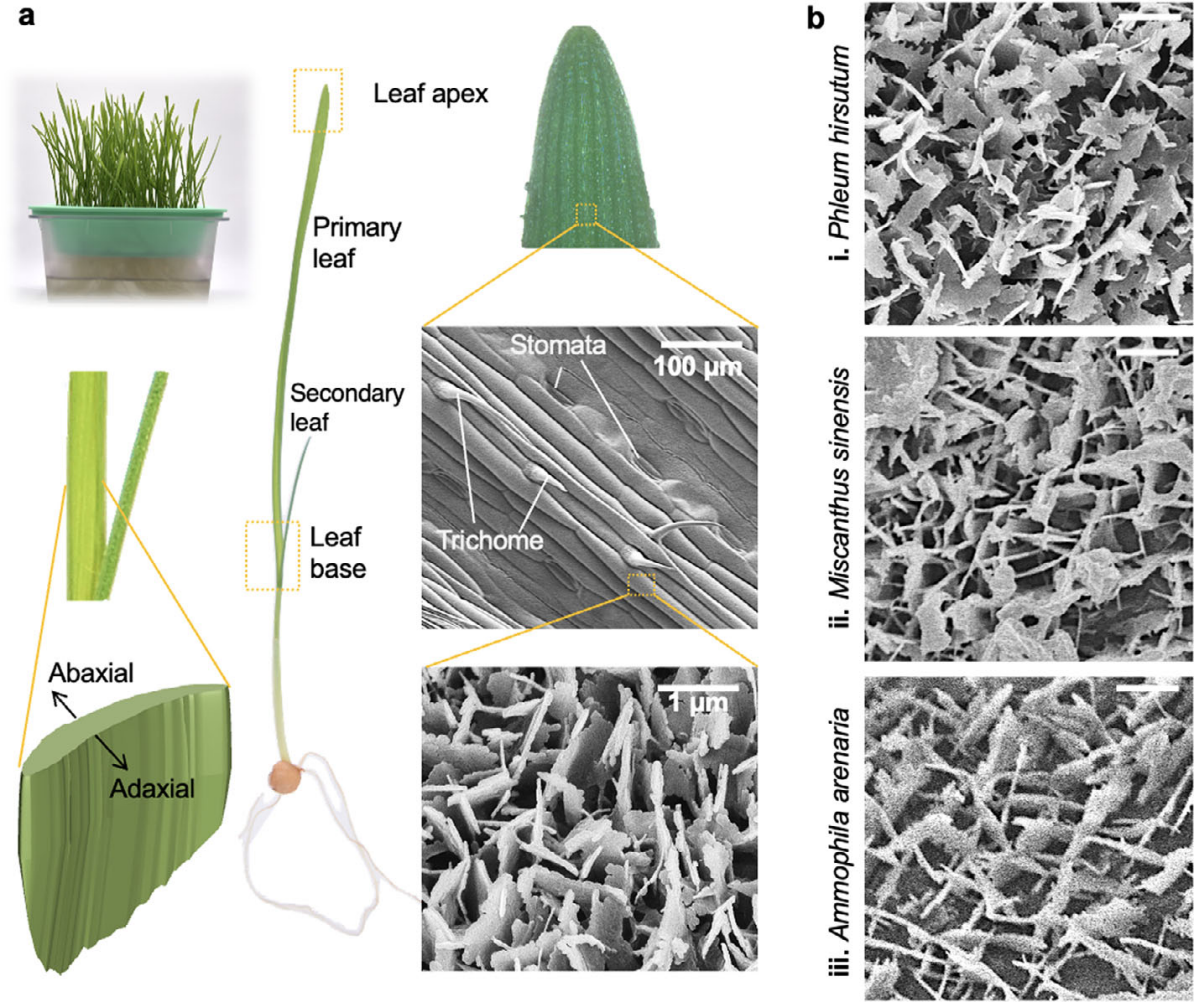
Key features of grass leaves used in this study. a) Images highlight key features of the wheatgrass plant, adaxial and abaxial sides, leaf regions and multiscale surface architecture. b) SEM images of epicuticular wax micromorphologies on grass leaves of i) *Phleum hirsutum* (2010‐1061), ii) *Miscanthus sinensis* (1969‐19098) and iii) *Ammophila arenaria* (1995‐1255), closely resembling the platelets found on the wheatgrass leaf. Leaves are harvested from the live collection at the Kew Grass Garden.

Here, we investigate several key aspects of the interfacial composition and architecture of natural leaves and their impact on the nucleation and growth of dew droplets at different environmental conditions, focusing on early stage microscale dynamics. Particular attention is paid to the impact of epicuticular wax micromorpholgy and coverage on the dynamics of dew droplets through performing in situ microscopy.^[^ ^66^ ^]^ Wheatgrass is chosen as a model organism since the physical micromorphology of the epicuticular wax on its leaves closely resembles that of other closely related^[^ ^67^, ^68^ ^]^ and distinctive grasses^[^ ^69^, ^70^ ^]^ (**Figure** **1**) as well as other plant families.^[^ ^51^, ^71^ ^]^ Furthermore, the wheatgrass leaf surface exhibits naturally consistent and reproducible variation in wax coverage across different regions, enabling analysis of the significance of wax distribution without the need for additional mechanical or chemical manipulation. Wheatgrass is also readily available, being easy to cultivate hydroponically under controlled conditions, thus facilitating systematic laboratory investigations.

We employ active cooling to allow systematic in situ investigations of condensation dynamics and correlate findings with the surface topography in different regions at the tip, centre and base along the leaves, distinguishing the abaxial and adaxial sides with identified variances in epicuticular wax coverage (Figure 1a). Wax‐based micro and nanoarchitectures are easily reproducible in a variety of hard, soft, and porous materials,^[^ ^52^, ^67^, ^72^, ^73^ ^]^ thus the mechanistic understanding of their performance in biological organisms provides opportunities for the design and fabrication of a new generation of sustainable dew harvesting coatings.^[^ ^6^, ^18^, ^23^ ^]^ Furthermore, the dynamics of leaf surface interactions with environmental water resources discussed here are closely related to the adsorption of water and liquid pesticides on plant surfaces^[^ ^74^, ^75^ ^]^ as well as disease transmission routes previously identified in wheat through jumping droplets,^[^ ^53^ ^]^ highlighting the relevance of this research to both natural and agricultural ecosystems.^[^ ^15^, ^41^, ^76^ ^]^

## 2 Results and Discussion

The formation of dew on grass leaves in their natural setting occurs as a result of effective passive radiative cooling during the darker hours.^[^ ^6^ ^]^ To perform a quantitative systematic analysis of dew dynamics under well‐controlled thermodynamic conditions, we performed measurements through active cooling of wheatgrass leaves using a Peltier device.^[^ ^77^ ^]^ This approach allows us to investigate the role of surface topography in isolation from variant environmental conditions. We report results of condensation experiments at subcooling levels in the range of Δ*T*_c_ = 2 − 10 °C, comparable to the observations in natural environments and reports of recent emerging engineering technologies.^[^ ^6^, ^18^, ^31^, ^53^, ^78^ ^]^

### 2.1 Surface Topography

**Figure 2:**
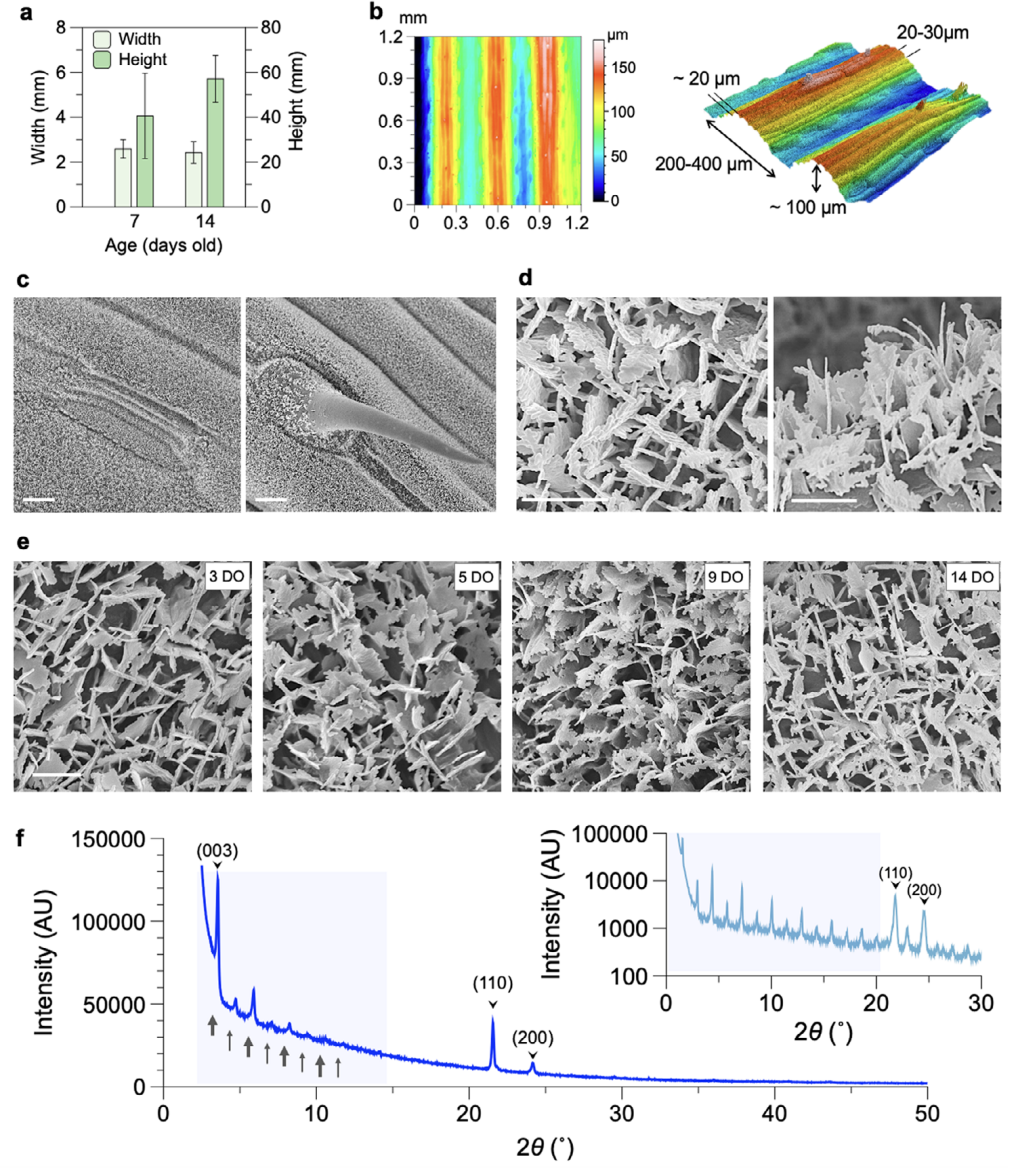
Multi‐scale topography of wheatgrass leaf. a) The average height and width, measured for leaves up to 7 and 14 days of age. Error bars represent the standard deviation of the population. b) 2D contour plots and 3D LSCM images show distribution of elevation across an example 1.2 x 1.2 mm^2^ region of the leaf. c) The adaxial surface of the leaf, including the stomata (left), is densely covered by the wax crystal plates, except for the trichomes (right). d) SEM close‐up images of the wax crystals show their standing platelets structures with ragged sides resembling a 2D fir tree. e) SEM images on the adaxial apex regions of leaves at different ages. Scale bars correspond to 10 µm in **c** and 1 µm in **d** and **e**. (f) X‐ray diffraction intensity for the wax collected from the leaf through dissolution in chloroform showing the orthorhombic crystal structure. Inset shows the diffraction intensity measured for pure 1‐Octacosanol powder. Small‐angle regions are highlighted in blue.

The surface of wheatgrass leaf embodies a combination of multi‐scale directional and isotropic topographies, ranging from a few millimeters down to nanometers (Figures 1 and **2**). The fresh wheatgrass leaves of ≈2 mm width grow predominantly in length, reaching an average height of 40 to 60 mm from 7 to 14 days, respectively (Figure 2a). Longitudinal veins and cell arrangements create compound surface undulations with dimensions of 10–100's µm, see Figure 2b and Figure S1 (Supporting Information).^[^ ^79^, ^80^ ^]^ At a significantly smaller scale, interfacial nano/micromorphologies appear as a result of molecular self‐assemblies and microscale aggregation of epicuticular waxes, central to the focus of this work.

The adaxial surface of the leaves, including the stomata, is covered by densely packed wax crystals (Figure 2c, left); the only exception is the trichome surface (Figure 2c, right) which exhibits decreasing wax crystal density from the base to tip. The wax micromorphology appears as randomly oriented platelets of ≈50 nm thickness with ragged edges similar to those observed on the mature leaf blades of *T. aestivum* (Figure 2d).^[^ ^67^, ^71^ ^]^ These platelets are 0.5–1 µm long and 0.5–2 µm high, and generally appear to be taller and more densely packed compared to those found on mature wheat (*T. aestivum*) blades.^[^ ^67^ ^]^ The wax crystals were observed to be well developed shortly after the emergence of the primary leaf. Starting from 5 days of leaf growth, randomly oriented wax platelets formed a populated hydrophobic mesh on the leaf, see Figure 2e. Most of the analysis presented here was therefore performed on freshly harvested 7‐day‐old blades with consistent wax coverage. No significant variation in wax coverage and crystal morphology was found in the apex region of the leaves of different heights harvested at a specific age, see Figure S2 (Supporting Information). The micromorphology of wax platelets on the surface of wheatgrass closely resemble the counterpart in other grasses from varied climates (Figure 1).

X‐ray diffraction analyses of the extracted wheatgrass leaf wax demonstrated the orthorhombic structure of the wax crystal (Figure 2f), manifested by the two intense head‐group spacing peaks at 4.1 and 3.7Å see Figure S3a (Supporting Information). Epicuticular wax platelets extracted from wheat blades were previously found to be mainly composed of primary alcohols, in particular 1‐octacosanol, which accounts for up to 70% of the total mass composition.^[^ ^67^, ^81^ ^]^ XRD measurement of 1‐octacosanol is provided as an inset in Figure 2f for comparison. The highlighted long‐spacing region (2θ ⩽ 20°) of the graph demonstrates equally spaced peaks at alternating high and low intensity, which were attributed to the alkyl‐alkyl and hydroxyl‐hydroxyl boundaries in the bilayer structure of the long‐chain fatty alcohols.^[^ ^67^ ^]^ Further analysis of the long‐spacing peaks confirmed a relatively wider bilayer d‐spacing in the extracted wheatgrass wax crystals compared to the pure 1‐octacosanol, possibly due to the existence of longer chain aliphatic compounds in the extracted wax, see Figure S3b (Supporting Information).

### 2.2 Surface Wetting

**Figure 3:**
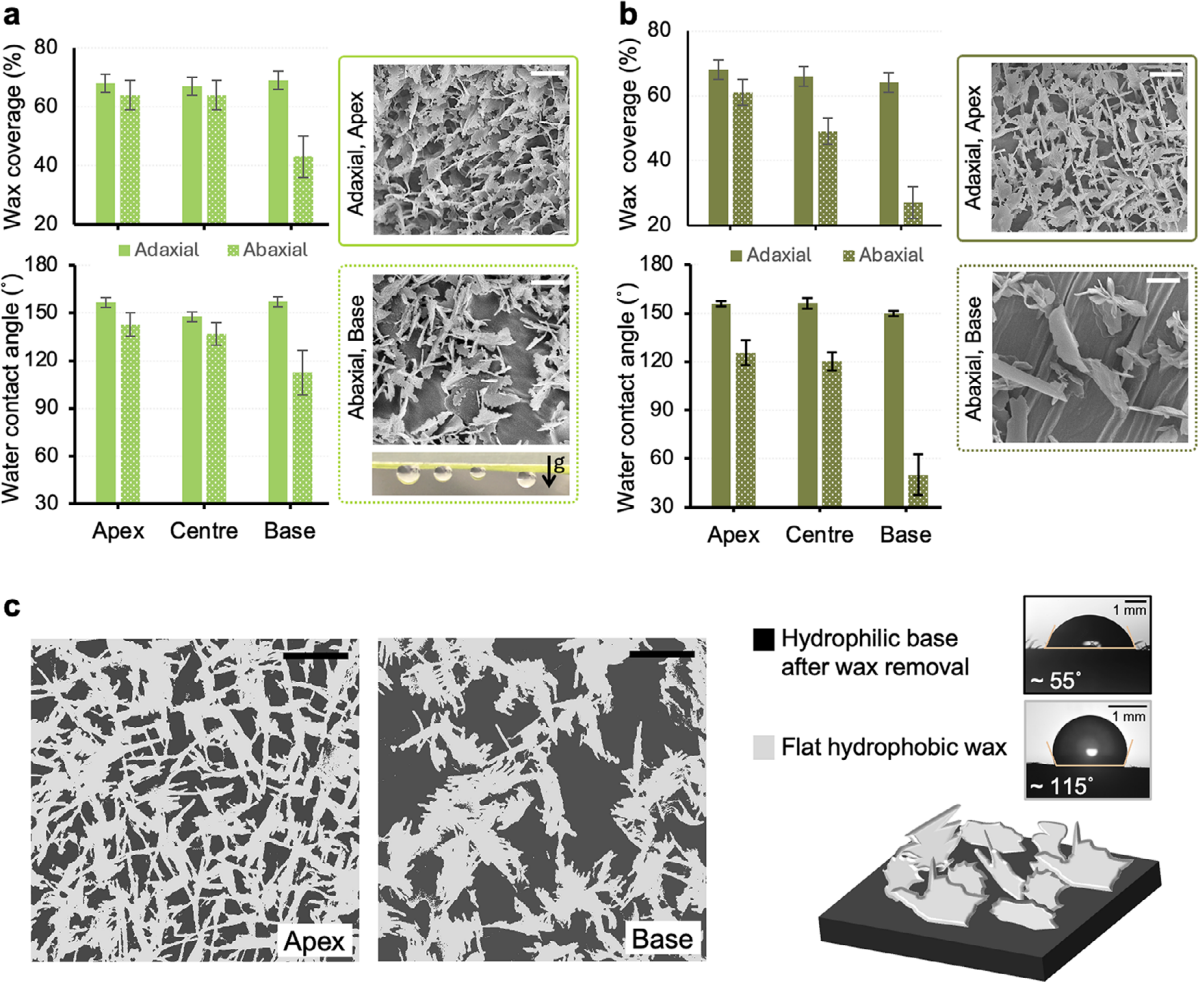
Impact of epicuticular wax coverage on the wetting behavior of the wheatgrass leaf. Wax surface coverage, water contact angle and representative SEM images across different regions on the adaxial and abaxial sides of a) freshly harvested 7 days old and b) dehydrated 14 days old leaves. Wax coverage is roughly estimated from the top‐view SEM images. Measurements are presented on the apex, centre and base of the wheatgrass leaves. The surface of the abaxial side is typically sticky toward water droplets. Error bar represent the standard deviation of average measurements on 5 independent leaves. Scale bars correspond to 1 µm in all images in **a** and **b**. c) Binary SEM images of the apex and base regions on the abaxial side of the 7 days old fresh leaves. Hydrophilic base and hydrophobic waxes are demonstrated in dark and light colors. Average contact angles are measured using micro‐imaging on the hydrophilic base after the removal of the epicuticular wax and a flat 1‐octacosanol coating, respectively.

Noticeably different wetting behavior was observed across different regions of the wheatgrass leaf, captured via static water contact angle measurements. The comparison between the epicuticular wax coverage and the measured water contact angle for the adaxial and abaxial sides is presented in **Figure** **3**a,b for freshly harvested and dehydrated leaves, respectively. The microsculptures on the adaxial side of the leaves yield slippery superhydrophobic properties with static water contact angles ⩾150°, similar to observations reported for other plant species.^[^ ^46^, ^67^ ^]^ In contrast, the wetting behavior varies from the apex to the base region on the abaxial side of the leaves; the region at the base of the leaves is less hydrophobic showing a decrease in contact angle values in both fresh (Figure 3a) and dehydrated leaves (Figure 3b). This behavior was found to be directly correlated with the epicuticular wax surface coverage. Regions with wax coverage above 65% exhibit superhydrophobic properties, while a significant drop in wax coverage from ≈60% to less than 30% caused a continuous increase in surface wetting from the tip to the base region on the abaxial side. In addition to reduced hydrophobicity, the base regions demonstrated a sticky wetting behavior with highly pinned droplets, as indicated in the inset image of Figure 3a.

The reduced hydrophobicity in the base region was more significant in the aged dehydrated leaves with more aggregated and dispersed wax crystal platelets, leaving extended wax‐free regions on the surface and resulting in significantly lower static contact angles ⩽90°. While long‐term exposure to drought in natural environments may cause changes in wax composition and often an increase in the cuticular wax content to minimize water loss in plants,^[^ ^82^, ^83^ ^]^ the reduction in the epicuticular wax coverage in the lab‐grown aged dehydrated leaves may be due to environmental mechanical abrasion or other aggregation mechanisms triggered by water loss and cell shrinkage in the leaf cuticles.^[^ ^67^, ^84^ ^]^ The considerable non‐homogeneity in the epicuticular wax crystal coverage resulted in significant pinning of the air‐water contact line in the wax‐free spaces, reported on other natural and laboratory‐made coatings of heterogeneous chemical or physical texture.^[^ ^85^, ^86^, ^87^ ^]^ A slight reduction in epicuticular wax crystal coverage was also observed in all regions of the adaxial side in aged dehydrated leaves, however, the consequences on the overall macroscopic wetting behavior of the surface was not significant (Figure 3b).

The enhanced surface wettability in regions with low wax coverage suggests a biphilic composition as a result of the hydrophilic leaf surface decorated with superficial hydrophobic wax platelets, as illustrated in the top‐view SEM images and the schematic in Figure 3c.^[^ ^88^ ^]^ Optical microscopy images in Figure 3c show contact angle measurements on the bare leaf (≈55 ± 5°) after wax removal and flat coatings made up of 1‐octacosanol (≈115 ± 2°), corroborating the biphilic properties of the surface of the wheatgrass leaf. Materials with such biphilic wetting behavior often perform efficiently as dew collectors in natural and engineered solutions.^[^ ^89^, ^90^ ^]^ As nucleation and growth of condensation droplets on surfaces with heterogeneous wetting properties are influenced by the dimensions and arrangement of wetting and non‐wetting features, we investigated the dew dynamics in leaf regions with distinctive wax coverage guided by our wetting analysis.^[^ ^91^ ^]^

### 2.3 Dew Dynamics

Dew formation on the wheatgrass leaf is an example of heterogeneous condensation on a rough biphilic surface. Hydrophilic substrates exhibit lower energy barriers to condensation nucleation. Continuous nucleating sites spread, however, resulting in the film‐wise condensation regime on such surfaces that is undesirable to both cooling and water harvesting applications.^[^ ^92^ ^]^ Hydrophobic interfacial microstructures are essential for the thermodynamic phase change to result in the formation of isolated quasi‐spherical droplets, referred to as drop‐wise condensation or breath figure formation.^[^ ^36^ ^]^ While the overall transition between the film‐ to drop‐wise condensation can often be simply predicted based on the macroscale wetting properties of the surface and the thermodynamic supersaturation level, dynamics of condensation growth is governed by interfacial interactions at micro‐ and nanoscale. We performed a series of condensation experiments on different regions of leaves with varying wax coverage at discrete saturation levels established by horizontally mounting leaf sections on a Peltier device at a set subcooling level Δ*T*_c_. The wax‐free elongated trichomes (Figure 2c) are expected to play a significant role in defining the fate of dew droplets only in later stages of condensation (Figure S4, Supporting Information), therefore, the present study of nucleation and growth in earlier stages focuses primarily on the micropatterned leaf lamina.^[^ ^62^, ^63^ ^]^ The following results highlight the role of epicuticular wax micromorphology in prescribing the outcome of condensation on the leaves and the establishing complex dynamics that is often engineered on deliberately designed nanostructured surfaces.

#### 2.3.1 Condensation Regimes

Tracking the average greyscale intensity in top‐view microscopy images after subtracting the background (initial reference image) offers a facile approach to visualize the ensemble effects of the nucleation and growth of dew droplets as well as different regimes of growth and coalescence. In situ bright‐field microscopy was performed in reflection mode, thus easily capturing the formation of curved microdroplet interfaces as the intensity of the grayscale increased in the image sequence.

**Figure 4:**
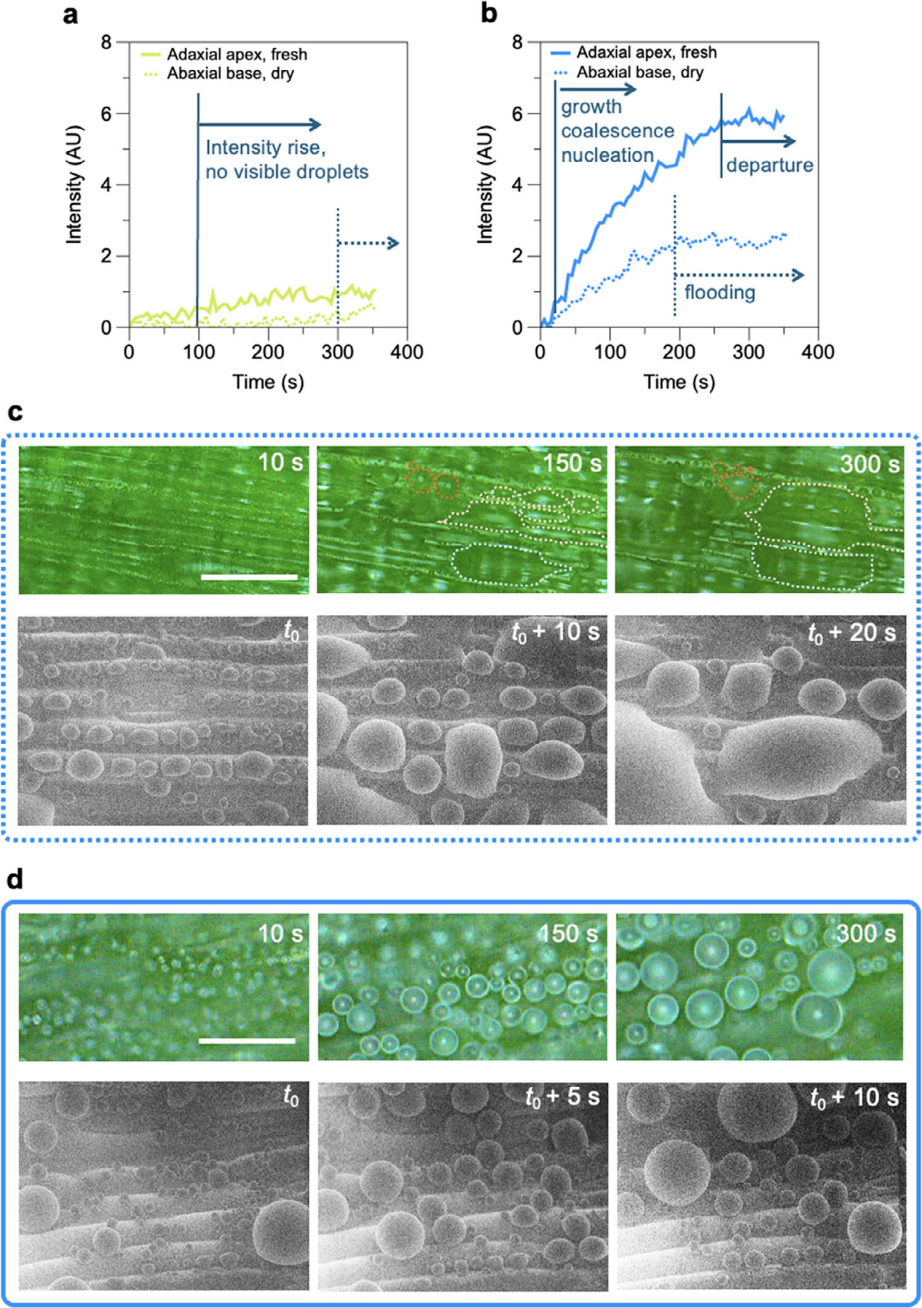
Condensation regimes on regions of varying hydrophobicity on wheatgrass leaves. Graphs show distinctive relative greyscale intensity evolutions at constant subcooling at a) Δ*T*_c_ = 2 °C and b) Δ*T*_c_ = 10 °C on the adaxial apex and abaxial base regions of fresh and dehydrated leaves. (a) No optically resolvable drop‐wise condensation is found at small subcooling within the duration of the experiments. The onset of gradual increase in the intensity was observed at earlier times on the apex region of fresh leaves possibly due to moisture adsorption or the formation of sub‐micron droplets on the surface. (b) At larger subcooling, drop‐wise condensation is observed on both surfaces. Optical microscopy (top) and ESEM (bottom) images of drop‐wise condensation evolution on the c) abaxial base and (d) adaxial apex of the wheatgrass leaves. Scale bar refers to 50 µm in optical and ESEM images in **c** ad **d**.

The example results of nucleation and growth of condensation droplets are presented in **Figure** **4** for the most disparate variations of the wax coverage regions between fresh and dehydrated leaves. The apex region of the adaxial side (fresh) is significantly more hydrophobic than the base of the abaxial side in dry leaves due to the higher intensity of wax coverage, with water contact angles measured at ≈155 °C compared to 45 °C from Figure 3. The graphs in Figure 4a and Figure 4b compare the results of nucleation at smaller (Δ*T*_c_ = 2 °C) and larger (Δ*T*_c_ = 10 °C) subcooling for these samples. For Δ*T*_c_ = 2 °C, no clear air‐water interface or droplet formation was observed regardless of the overall surface wetting characteristic (Figure 4a). Environmental SEM analysis at small subcooling showed a rare appearance of unstable small condensation droplets that immediately disappeared. In contrast, the two regions on the abaxial and adaxial sides of the leaves promoted different regimes of condensation at larger subcooling. The initial drop‐wise condensation on the base regions spreads on the more hydrophilic surface, generating larger flooded regions (Figure 4c) manifested by an extended region of constant intensity in Figure 4b, see Video S1 (Supporting Information). Relatively larger number of spherical micro droplets were found on the superhydrophobic adaxial side of the leaf tip, with regular coalescence upon growth (Figure 4d), see Video S2 (Supporting Information). These droplets maintained their spherical shape from the early stages of condensation throughout the growth regimes (Figure S5, Supporting Information). Droplet coalescence reduced the overall number of droplets, leading to a slower increase of captured reflected light intensity at intermediate times. This resulted in a quasi‐constant intensity regime (Figure 4b), where coalescence results in the departure of a significant number of droplets from the surface, which were subsequently replaced by a new generation of small droplets.^[^ ^93^ ^]^

Three overall distinct condensation dynamic regimes across the different regions of wheatgrass leaves are observed, captured through the ensemble relative grey‐scale analysis: i) moisture adsorption with no detectable air‐water interface formation at smaller subcooling (Δ*T*_c_ = 2 °C) across all analyzed samples, ii) drop‐wise condensation leading to non‐spherical growth, interface pinning and local flooding in less hydrophobic abaxial base regions, and iii) nucleation and spherical growth of dew droplets in superhydrophobic regions. Increasing the subcooling level induced larger initial nucleation densities and droplet growth rates, resulting in significant droplet coalescence and departure in (iii). Overall, the biphilic wetting property of the surface quantified in Figure 3 supports the presence of identified condensation regimes; while dew nucleation was observed on all regions on the adaxial and abaxial sides, a greater wax coverage ⩾65% is essential for effective transformation to drop‐wise condensation in early stages of the process.

#### 2.3.2 Kinetics of Drop‐Wise Condensation

**Figure 5:**
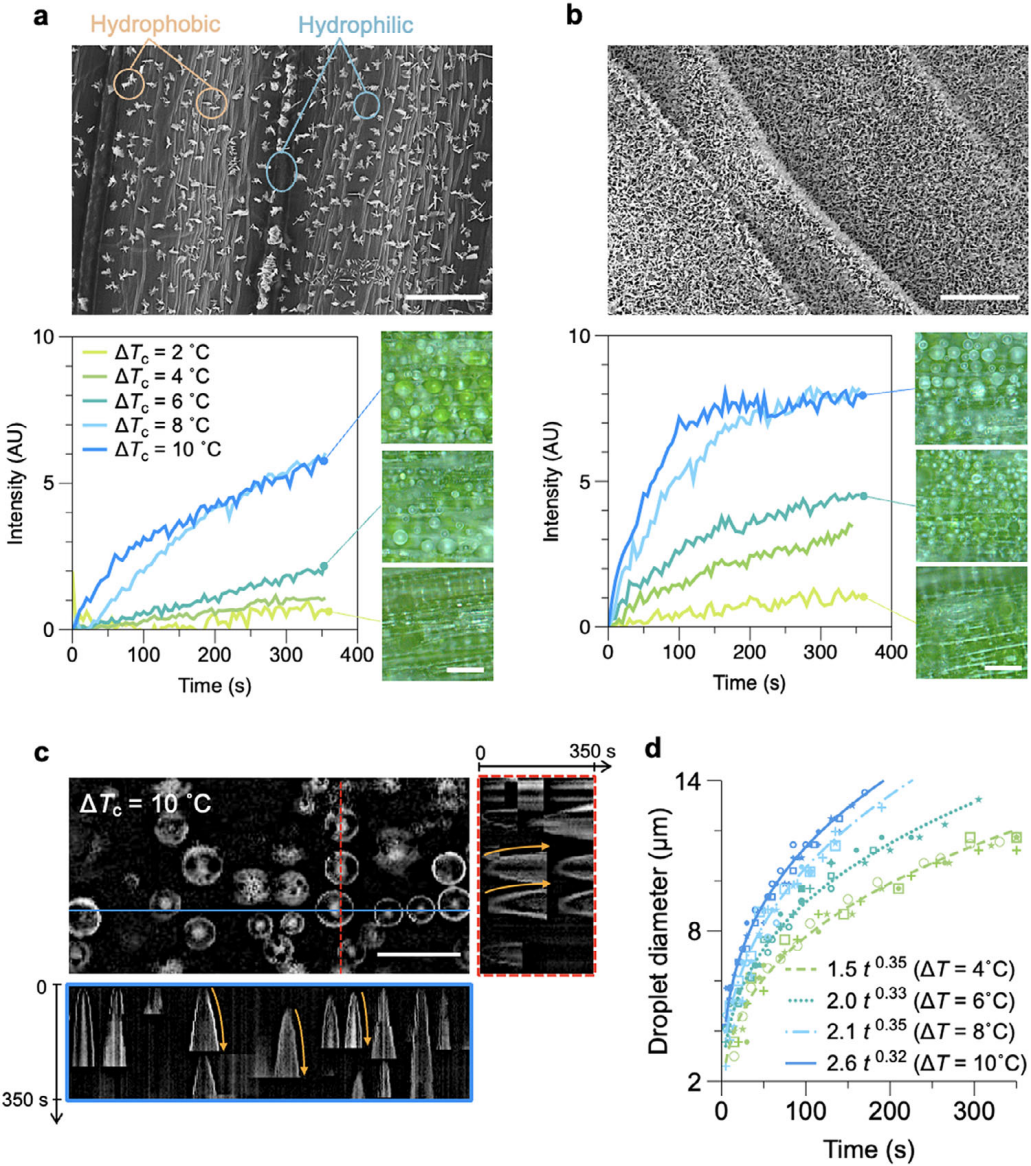
Impact of wax crystal coverage on the nucleation and growth of condensation droplets on the abaxial surface of fresh 7 days old leaves. SEM images show sparse wax crystals on the base a) relative to the higher wax coverage on the apex region b). Graphs show the relative mean greyscale intensity evolution during subcooling experiments at varied subcooling level denoted by Δ*T*_c_ for the base and the apex regions. Optical microscopy images refer to the final state in the cooling experiment at 350 s. c) Orthogonal time‐strip analysis demonstrates the symmetric growth of condensation droplets on the apex region by tracking the relative greyscale intensity along horizontal and vertical lines in a cropped view of microscopy images. Arrows show the direction of growth along the detected bright interface of the droplets. d) Growth of individual droplets on the apex region (**b**, **c**) is governed by molecular diffusion before coalescence occurs, captured by a power‐law relationship between the droplet and time. Scale bars refer to 10 µm for SEM images in **a** and **b**, and 50 µm in optical micrographs presented in **a, b** and **c**.

**Nucleation and Growth**. While the adaxial surface of the fresh leaf remains superhydrophobic throughout with approximately constant wax coverage, the epicuticular wax distribution on the abaxial region varies throughout the blade length showing a consistent increase in surface coverage from the base to the tip. **Figure** **5**a,b highlight comparative view of the wax coverage variation and subsequent nucleation and growth on the abaxial base and apex regions of fresh 7 day old leaves, respectively. Video S3 (Supporting Information) provides a side‐by‐side cropped view of these microscopy data.

Drop‐wise nucleation and growth was observed on both abaxial regions for Δ*T*_c_ ⩾ 4 °C throughout the analysis, however, the number of detected microdroplets was significantly smaller on the base region with dispersed wax crystal aggregates (Figure 5a) compared to the uniformly coated apex region (Figure 5b). The base region exhibited droplets that grew into larger non‐spherical shapes, resulting in an overall lower detected light reflection intensity in the microscopy images. While the hydrophilic base promoted nucleation, the denser packing of hydrophobic wax crystals was essential for the growth of a larger number of spherical droplets, yielding overall larger intensities detected on the apex region. The overall uniformity of wax coverage on the apex further enhanced droplet coalescence and mobility, reflected in the plateau region in intensity‐time graph at later times for higher levels of subcooling (Figure 5b). This is similar to the full region of the adaxial side, where due to high wax density, comparative nucleation and growth dynamics and observed.

Time‐strip analysis captured the growth and coalescence dynamics of spherical dew droplets, where the time evolution of relative grey‐scale intensities is tracked along orthogonal lines (Figure 5c). The growth of droplets across the two orthogonal axes lead to the appearance of horizontal and vertical cones that expand over time. The interruption in the growth of the cone base diameter is due to the coalescence of neighboring droplets and the dislocation or departure of the newly formed droplet. The measurement of the in‐focus single droplet diameter in time follows the well‐known power law *D*∝*t* ^1/3^ predicted for the growth of sessile condensation droplets dominated by molecular diffusion (Figure 5d).^[^ ^94^, ^95^ ^]^ Considering the optical resolution of our microscopy setup, the uncertainty in droplet diameters presented in Figure 5d was within 15% of the reported values. Longer periods of diffusion‐dominant growth were captured for the lowest subcooling level (Δ*T*_c_ = 4 °C) as a consequence of the lower initial nucleation density and coalescence, and slower droplet growth.

**Figure 6:**
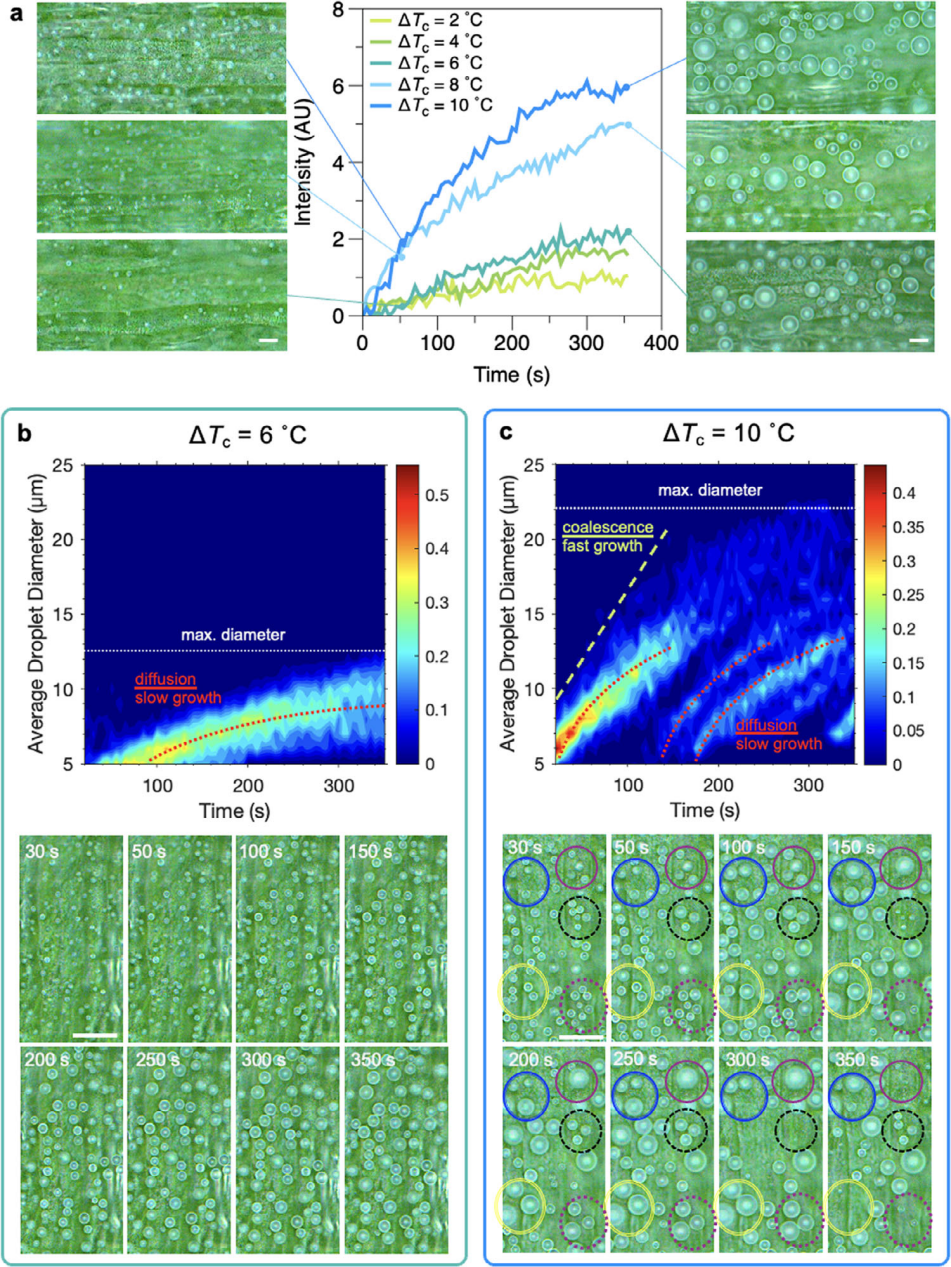
Dew formation on the adaxial apex region of the 7 days old wheatgrass leaves. a) Evolution of greyscale intensity in time at different levels of subcooling. Micrographs correspond to cropped regions of the original images at 50 and 350 s. b) Population density of average droplet diameter at Δ*T*_c_ = 4 °C highlighting the diffusion dominated growth regimes. c) Growth, coalescence, departure and recondensation events during a subcooling experiment at Δ*T*_c_ = 10 °C. Droplet coalescence and departure enables re‐condensation appearing as new populations of droplets growing through molecular diffusion between the coalescence events. Circles highlight regions of the image where droplet departure and re‐condensation occur. Scale bars correspond to 20 µm in all microscopy images.

**Coalescence and Departure**. On the superhydrophobic regions with uniform wax coverage at the abaxial apex and the entire adaxial side of the leaves, drop‐wise condensation was observed for Δ*T*_c_ ⩾ 4 °C. Nucleating droplets were immediately transported onto the wax microplatelets in a suspended Cassie–Baxter regime.^[^ ^96^ ^]^ As a result, spherical droplets were formed from the early stages of optical microscopic detection (diameter ⩾ 3 µm) on the adaxial apex region of the leaves (Figure S5, Supporting Information). On these superhydrophobic regions of the leaves, the subcooling temperature was found to significantly impact the initial nucleation density, growth and thus the probability of droplet coalescence events (**Figure** **6**). While the hydrophilic base promoted nucleation of droplets, the hydrophobic wax platelets ensured formation of highly mobile spherical droplets even at low subcooling levels; see inset images in Figure 6a. These droplets were observed to grow symmetrically in time and coalesce frequently, captured by the appearance of a plateau region in the grey‐scale intensity for subcooling levels higher than Δ*T*_c_ = 4 °C (Figure 6a). Analysis of population density of droplet diameter was performed over cropped in‐focus regions of the images to quantitatively describe aspects of the condensation dynamics (Figure S7, Supporting Information).

At lower subcooling level Δ*T*_c_ = 4 − 6 °C, the grey‐scale variation increases at a much slower rate compared to larger subcooling, Δ*T*_c_ = 8 − 10 °C (Figure 6a). Inset images in Figure 6b for Δ*T*_c_ = 6 °C show isolated droplets growing almost exclusively via diffusion of water vapour from the air. Little to no coalescence between neighbouring droplets occur within the experimental time frame. The time evolution of droplet diameter density in Figure 6b demonstrates this with a concentrated narrow range in the average diameter over time mainly governed by diffusion mechanism, described by *D*∝*t* ^1/3^.^[^ ^94^ ^]^ Slower droplet growth and limited coalescence at moderately small subcooling levels restrict the average and maximum droplet diameters at ≈9 and 12 µm, respectively.

At higher subcooling level (Δ*T*_c_ = 8 − 10 °C), both number density and growth rates of condensation droplets increased significantly, leading to frequent coalescence of the droplets captured by the plateau region in the grey‐scale intensity graph after ≈200 s from the start of the experiments, demonstrating that a balance between the old interfaces disappearing and newly generated interfaces is achieved. Figure S8 (Supporting Information) compares examples of droplet dynamics at smaller and larger subcooling through time‐strip analysis. The plot of diameter population density versus time shows a much faster initial droplet growth over the first 150 s, followed by multiple new families of droplets being nucleated (compare Figures 6b with 6c); the coalescence of the droplets was captured as an increase in the slope and a widening of the intensity‐time curves.^[^ ^77^, ^95^ ^]^ A close‐up view of the microscopic images of the condensation further links quantitative light intensity measurements to the kinetics of nucleation and growth of condensation droplets.

At the highest subcooling level tested here, microscale droplets were observed to nucleate across the microstructured surface and readily coalesce upon contact with neighbours (Figure 6c). Between the 100 and 150 s time frames, coalescence events between multiple droplets and high mobility led to the energetic departure from the surface, freeing up space for the nucleation of new droplet families (Videos S2 and S4, Supporting Information). Two distinct regimes of droplet growth are detectable in the temporal evolution of droplet diameter populations at lower subcooling temperatures presented in Figure 6c; isolated droplets follow the diffusion dominated regime of *D*∝*t* ^1/3^, while regular coalescence accelerates the growth to a linear regime *D*∝*t* ^1^.^[^ ^94^ ^]^ An average pre‐coalescence departure radius of 7.7 ± 1.3 µm was quantified for the data at lower subcooling temperatures (Figure 6c). Due to the larger initial nucleation density and high mobility of the droplet interface on the nanostructured adaxial surface of the leaf, coalescence typically occurred between two or three droplets leading to jumping droplets of ≈10 µm radius within the working time covered in our experiments. This is comparable to some of the most efficient engineered surface designs for continuous drop‐wise condensation and shedding (Figure S7, Supporting Information),^[^ ^93^, ^97^, ^98^, ^99^, ^100^, ^101^, ^102^, ^103^, ^104^, ^105^ ^]^ and is in line with findings reported previously on jumping dew droplets identified as a pathways for self‐cleaning and disease transmission in plants.^[^ ^53^, ^55^ ^]^ The effective dynamic nucleation, growth, coalescence, departure, and re‐nucleation cycle on wheatgrass keeps the maximum detected diameter below ≈20 µm within the experimental time frame analysed.

**Figure 7:**
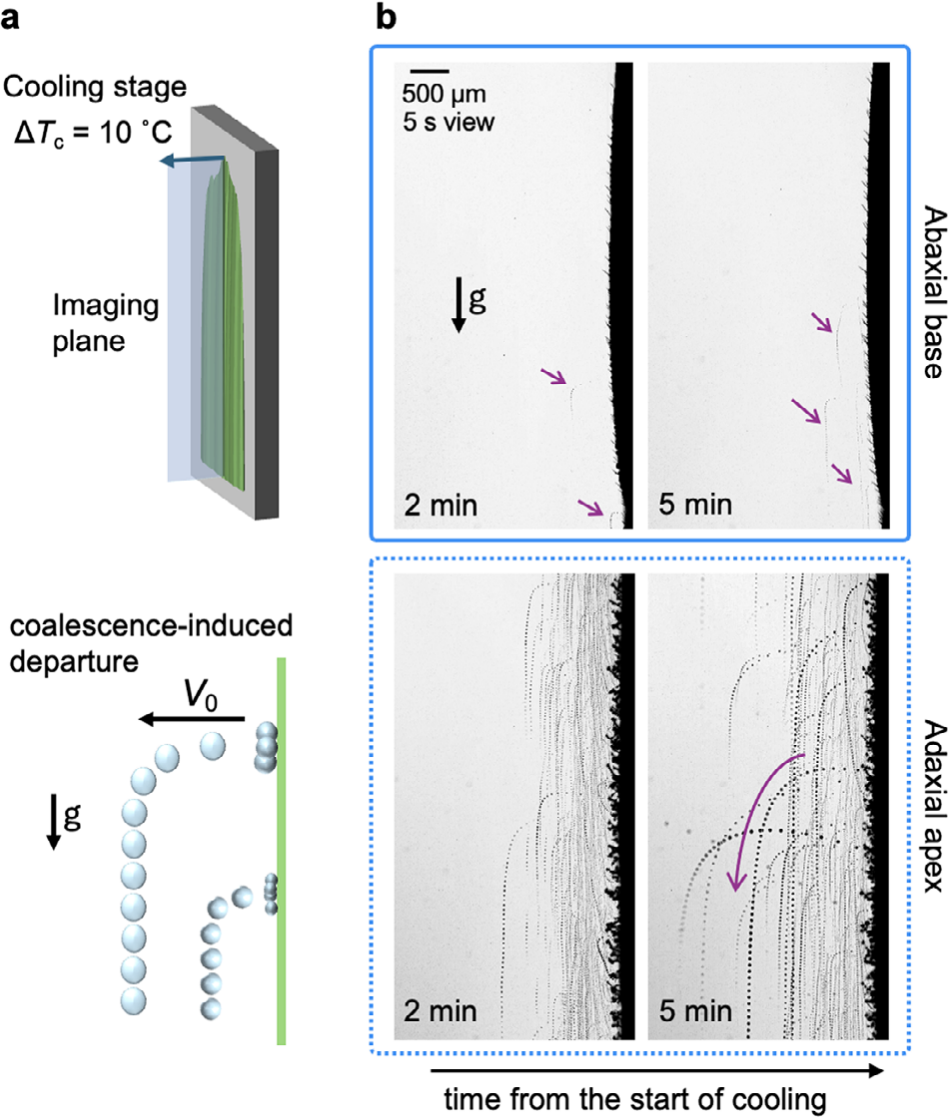
Dew formation on a vertical wheatgrass blade. a) Wheatgrass leaves are mounted on a vertically held Peltier cooling device and chilled down to achieve 10 °C of subcooling. Side‐view high‐speed imaging of the nucleation dynamics at 1000 fps allows tracking of jumping dew droplet trajectories. b) On the less hydrophobic, sticky surface at the base of the abaxial side of the leaf, jumping droplet were rarely observed within 5 min experimental time frame. On the superhydrophic apex region of the adaxial side, frequent droplet jumping events were record after 100's s from the start of the experiments. High‐speed imaging at later time (5 min) captured more frequent jumping events and appearance of larger droplets. Presented images show composite trajectories captured over 5 s.

In situ optical analysis of horizontally oriented specimens facilitates top‐view microscopic imaging, providing information on the dew formation regimes regarding the size and number of coalescing droplets. However, grasses, particularly among short‐statured species, predominantly exhibit a vertical or near‐vertical orientation in natural environments, resulting in varying jumping dynamics due to the action of gravitational forces. A more natural configuration was hence modeled on vertically orientated leaf samples to interrogate the out‐of‐plane jumping dynamics of micro dew droplets. We implemented an alternative experimental setup employing an identical Peltier cooling module to replicate subcooling conditions comparable to those used in the horizontal configuration, using a high‐speed shadowgraphy imaging set‐up recording at 1000 fps (**Figure** **7**a). In line with the top‐view observations presented in Figures 4 and 6, jumping of coalescing jumping droplets was rarely observed during the 5 min experimental run time on the abaxial base. On the adaxial side, fast micro droplets of diameters 10–30 µm were observed to frequently depart the surface at initial speeds of *V*_0_~ = 0.3–0.5 m.s^−1^, reaching up to 2–3 mm away from the leaf surface, corroborating previous findings on winter wheat leaves.^[^ ^53^ ^]^ Based on typical high‐speed imaging results considered over a period of 5 s at 5 min from the start of cooling (Video S5, Supporting Information), we estimate ≈ 5 mg cm^−2^.h fresh water collection in the form of free falling micro dew droplets from the adaxial side of a single grass blade. An average diameter of 15 µm for an approximate hundred optically detectable jumping events was considered over a 9 × 2 mm^2^ area of interest for this estimate. An average density of 10–20 shoots per cm^2^ of ground surface is expected in short grasses depending on the plant architecture. Therefore, the overall dew amount over the plant footprint area can be 10–20 times larger, yielding up to 50–100 mg cm^−2^.h collected water that is comparable to modern small and large‐scale dew harvesting solutions.^[^ ^9^, ^89^, ^90^, ^106^ ^]^ The stationary collected dew on the less hydrophobic abaxial side of the wheatgrass may serve as an additional source of water accessible through foliar uptake.

## 3 Conclusion

The diversity in design and habitats of grasses makes them an ideal model for discovery‐led research investigating the link between architecture and environmental function. In this study, we investigated the microscale interfacial structure on wheatgrass leaves and the impact of its composition, morphology, and distribution on the formation and early stage dynamics of dew droplets. By investigating the role of hydrophobic epicuticular wax crystals in condensation, we identified regimes of moisture adsorption and dew formation on the surface of wheatgrass leaves as a representative model. The epicuticular wax, made up of orthorhombic crystal structures, generated interfacial hydrophobic micromorphologies in the form of extended platelets of approximate order of 10's nm thickness and 1's µm length/height on the hydrophilic foundation of the leaf, resulting in biphilic wetting properties. On the abaxial side, the wax coverage reduced from the apex region toward the base of the leaf, resulting in reduced hydrophobicity and local transitions from drop‐wise to film‐wise condensation. Regions of high wax coverage on the adaxial side of the leaves show slippery superhydrophobic properties that promote effective drop‐wise condensation through continuous cycles of nucleation, growth, coalescence, departure, and re‐nucleation. The interfacial hydrophobic wax platelets hence appear as a promising surface treatment choice for the scalable fabrication of bioinspired functional coatings, benefiting from the availability and sustainability of waxes and the simplicity of available manufacturing techniques.^[^ ^99^, ^107^ ^]^ The example of biphilic grass leaf surface suggests that sustainable functional structured coatings can be developed through the previously introduced scalable plant‐sourced wax crystallization approaches on smooth and porous surfaces via vapour deposition or solvent evaporation.^[^ ^52^, ^72^, ^73^, ^108^ ^]^

By interrogating dew formation on the superhydrophobic areas of vertically orientated grass leaves, the coalescence of highly mobile droplets are observed to induce the rapid ejection of micro dew droplets as far as 2–3 mm away from the leaf. This observation provides new evidence that grass leaves may contribute a significant amount of fresh water supply to the soil and other organisms in the vicinity. The higher adhesion of water droplets on the abaxial was maintained in the vertical orientation possibly contributing to leaf water content during episodes of dewfall. Microscale surface features in the form of indented grooves or protruded trichomes are expected to have a significant effect on the later‐stage dynamics of dew formation and collection of the larger droplets on the actual grass blades, in addition to leaf deformability and edge effects. Future work on vertical standing grass blades will be essential to identify the fate of the dew droplets reported here on horizontal leaves. While microscale observations reported in this work are fundamental to understanding the ecological significance of dew formation on grasses, identifying the impact of reported trends on grassland microclimates, and the overall humidity regulation and water collection of the plant will require further comprehensive, interdisciplinary and multiscale studies that combine field observations with laboratory measurements.

## 4 Experimental Section

### Wheatgrass Leaf Growth

Seeds of wheatgrass, first leaves of the common wheat plant (*Triticum aestivum*), were purchased from Pretty Wild Seeds, UK. To optimize germination conditions, seeds were rinsed and allowed to soak for up to 3 days. The seeds were rinsed twice daily during the soaking period and then transferred to standard hydroponic sprouting trays before being placed in a growth chamber on day 4. The growth chamber was maintained at a relative humidity of 60% and a temperature of 20 ± 3 °C with a scheduled 16/8 h light/dark cycle to simulate natural daylight conditions and promote growth. The sprouting trays consisted of a mesh top where the germinated seeds were spread and a bottom reservoir tray in which roots grew; the water in the reservoir (root container) was changed twice daily in the morning and evening. Plant specimens were harvested at discrete growth stages shortly after the leaf emergence stage and up to 14 days after the initial germination conditions were started. This growth period is significantly shorter than that of wheat plant crops grown for human food production,^[^ ^67^, ^81^ ^]^ facilitating high throughput of fresh samples for analysis. Specimens were harvested immediately prior to analysis to ensure that the leaf blade remained in a hydrated state. Analyses were carried out in the apex, center and base regions of the primary leaf shoot, on both adaxial and abaxial sides (Figure 1). These regions of interest were isolated by dividing the total length of the leaf into three areas of equal length. Sections of 1 cm length were cut from the topmost (apex), central (centre) and lowest (base) parts of these regions of interest and used for all subsequent morphological and condensation analyses. Physical measurements of the grass leaves were taken at several stages of growth. The width and length of individual leaves were measured using a caliper gauge and a ruler. For dehydrated samples, wheatgrass leaves were removed from the growth chamber and left in a low‐humidity environment at *RH* ≈ 40% from day 10 to 14.

Leaves of grasses *Phleum hirsutum* (Catalog number: 2010‐1061) and *Miscanthus sinensis* (Catalog number: 1969‐19098) were harvested on 20/01/2025. *Ammophila arenaria* (Catalog number:1995‐1255) was harvested on 28/07/2025. All leaves were collected from live plants at Grass Garden, Kew, Richmond, UK.

### Surface Analysis

**Leaf Surface Topography**. Surface topography measurements were performed on fresh leaves using a Laser Scanning Confocal Microscope (LSCM) (Carl Zeiss LSM800) equipped with a 405 nm laser, with data exported to MountainsMap software (Digital Surf). Scanning electron microscopy (SEM) analysis was performed on freshly cut sections of leaves mounted on a stub using double‐sided carbon tape. The samples were sputter‐coated with 10 nm platinum prior to imaging on a Hitachi SU8230 microscope operated at 2 kV accelerating voltage.

**Wax Crystal Structure**. Crystal structures were analysed by X‐ray diffraction (XRD), using a Bruker D8 diffractometer with monochromatic Cu Kα radiation (2θ range 2.5°–50°, step size = 0.013°). Measurements were carried out over 12 h to improve the signal‐to‐noise ratio. The epicuticular wax was dissolved in chloroform by submerging the leaves in the solvent for 10 s. The solution was then filtered and drop‐casted on zero‐background silicon substrates. Powder X‐ray diffraction was also performed on 1‐Octacosanol (⩾ 99%) sourced from Sigma‐Aldrich.

**Wetting Analysis**. Fresh wheatgrass leaves were harvested before each measurement and mounted on microscope glass slides using double‐sided tape. Care was taken to ensure that the region of interest was not touched when securing the sample flat to the substrate. Static contact angle measurements were performed using a customized micro‐imaging setup and analyzed using the drop analysis plugin on ImageJ.^[^ ^109^ ^]^ The average value of the left and right static contact angles was measured for water droplets of 2 µm volume, with five independent leaves tested for each condition. On each surface, the average contact angle for three droplets was calculated to account for the surface nonhomogeneity. Droplet deposition by pipetting was not possible in the superhydrophobic sections of leaves; therefore, water droplets were injected using a syringe and held attached to blunt needles above the surface. The measurements were then performed by gradually raising the level of the leaf surface to contact the water droplets. The error bars on all presented graphs correspond to the standard deviations.

**Wax Coverage**. Top‐view SEM images were used to draw an estimate of the wax surface coverage in this study. SEM images taken from similar regions of three grass leaves were binarized and used for wax coverage analysis. The error bars on the graphs presented in Figure 3a correspond to the standard deviation of the measurements.

### In Situ Condensation Visualization

**Optical Microscopy**. Condensation experiments were performed under monitored laboratory environmental conditions of temperature (*T*_0_ = 24 ± 1 °C) and relative humidity (*RH* = 60 ± 5%). Fresh wheatgrass leaves were mounted on standard borosilicate glass cover slips of 24 mm × 24 mm with a thickness of 0.15 mm to ensure minimal thermal resistance. The mounted leaf was placed on the Peltier device held at a constant temperature *T*_P_ during each experiment, accurate to within ±0.5 °C. The subcooling temperature was calculated based on the difference between the measured dew point and the set Peltier temperature, Δ*T*_c_ = *T*_dp_ − *T*_P_. Condensation dynamics were monitored using an Olympus BX53M optical microscope (OM) in reflective mode, equipped with a long working distance objective (Olympus LMPLFLN 20X) and a digital CMOS camera (Basler ace acA2040‐90uc). All kinetics were recorded and analyzed starting from the initial time the grass leaf was exposed to the desired subcooling, capturing images every 5 s with a nominal spatial resolution of 0.55 µm.pix^−1^. Cropped in‐focus regions within the field of view were isolated for direct droplet diameter identification^[^ ^110^ ^]^ yielding a maximum uncertainty of 10% in the data presented in Figure 6. Five independent condensation analysis runs were performed for each location, using newly harvested leaves to ensure reproducibility. The graphs included in Figures 4, 5, and 6 of the article represent examples of various single runs at different locations and environmental conditions to improve visibility of the trends. Data analysis was performed in ImageJ on greyscale images by subtracting the initial image from the sequence and tracking the average greyscale intensity over time. The nucleation and growth of condensation droplets resulted in the appearance of higher greyscale intensity in the background‐subtracted images because of the high reflection at the water‐air interface. Although larger microscale surface topographies at the location of the veins produced out‐of‐focus effects across the images, quantifying the ensemble intensity of the reflected light allowed for effective tracking of the condensation kinetics from the nucleation and growth of new interfaces across the entire field of view.

In‐focus image sections of sample experiments that displayed drop‐wise condensation were analyzed by calculating the average droplet diameter. The images were digitally enhanced to increase the contrast of the droplet borders before running the previously developed MATLAB (Mathworks, R2024b) code to automatically identify droplet perimeters across the time series.^[^ ^110^ ^]^ The population density distribution of the average droplet size measured in time was visualized using 3D mesh plots and the corresponding contour plots. In each time frame, 1 µm bins were created with normalized counts to create a density distribution.

Side‐view imaging was performed using a Phantom Miro C210 high‐speed camera equipped with a Navitar 12x zoom lens providing an optical resolution of 7.0 µm.pix^−1^. A collimated white LED light source, Thorlabs MCWHL8, was used for back illumination. At this magnification, an approximate frame of reference of 9 mm of grass blade was observed with the full width (≈ 2 mm) in focus.

**Environmental Scanning Electron Microscopy**. Early‐stage nucleation and growth of water condensation droplets were imaged using FEI Quanta 650 with a tungsten source. Fresh samples were harvested prior to each analysis and mounted on angled conductive stubs placed on a Peltier module. No conductive coating was used to view the condensation kinetics on the virgin grass surface. The microscope chamber was purged and pumped down to 200 Pa before decreasing the Peltier temperature to 2 °C, ensuring the relative humidity remained well below the saturation pressure. The chamber pressure was slowly increased to increase the relative humidity, with images taken at different times using a 5 kV accelerating voltage.

## Conflict of Interest

The authors declare no conflict of interest.

## Supporting information


Supporting Information

Supplemental Video S1

Supplemental Video S2

Supplemental Video S3

Supplemental Video S4

Supplemental Video S5

## Data Availability

The data that support the findings of this study are available in the supplementary material of this article.
