# Supplementary material for: On the Formation and Dynamics of Micro Dew Droplets on Grass: the Role of Epicuticular Wax
Source: Small. 2025 Aug 21;21(39):e02219. doi: 10.1002/smll.202502219 (PMC12490185; doi:10.1002/smll.202502219)
Supplement: Supplementary file 1 — Supporting Information [file SMLL-21-e02219-s006.pdf]

NANO · MICRO
small

Supporting Information

for *Small*, DOI 10.1002/smll.202502219

On the Formation and Dynamics of Micro Dew Droplets on Grass: the Role of Epicuticular Wax

*Bashra Mahamed, Francis James Dent, Robert Simpson, Nicola Weston, Maria S Vorontsova, Fanny Nascimento Costa and Sepideh Khodaparast**

Supporting Information
On the formation and dynamics of micro dew droplets on grass: the role of epicuticular wax

Bashra Mahamed[1], Francis James Dent[2], Robert Simpson[3], Nicola Weston[4], Maria S Vorontsova[5], Fanny Nascimento Costa[6], and Sepideh Khodaparast[2*]

[1]School of Physics and Astronomy, University of Leeds, Leeds LS2 9JT, UK.
[2]School of Mechanical Engineering, University of Leeds, Leeds LS2 9JT, UK.
[3]School of Chemical and Process Engineering, University of Leeds, Leeds LS2 9JT, UK.
[4]Nanoscale & Microscale Research Centre (nmRC), University of Nottingham, University Park, Nottingham NG7 2RD, UK.
[5]Royal Botanic Gardens, Kew, Richmond, UK.
[6]The Bragg Centre for Materials Research, Sir William Henry Bragg Building, University of Leeds, Leeds LS2 9JT, UK.
[*]Address correspondence to: s.khodaparast@leeds.ac.uk

Supplemental videos

Video S1. Top view microscope analysis of flooding on the abaxial base of fresh 7 days-old wheatgrass leaves at $\Delta T_c = 8$ °C. Frame of reference is 165 µm wide.

Video S2. Top view microscope analysis of drop-wise condensation on adaxial apex of fresh 7 days-old wheatgrass leaves at $\Delta T_c = 8$ °C. Frame of reference is 165 µm wide.

Video S3. Comparative view of droplet growth on abaxial apex vs base regions of fresh 7 days-old wheatgrass leaves at $\Delta T_c = 8$ °C.

Video S4. ESEM growth of droplets on adaxial apex of fresh 7 days-old wheatgrass leaves at approximately $\Delta T_c = 10$ °C.

Video S5. High-speed shadowgraphy of a vertically orientated adaxial apex section of a fresh 7 days-old wheatgrass leaf. Video recorded at 1000 fps showing 1 s of real-time droplet jumping.

1 Leaf topography

The wheatgrass leaf is composed of various topographic characteristics; on a larger microscopic scale, the veins and cells generate compound undulations that are covered by smaller micro/nanoscale wax crystals (Fig. S.1).

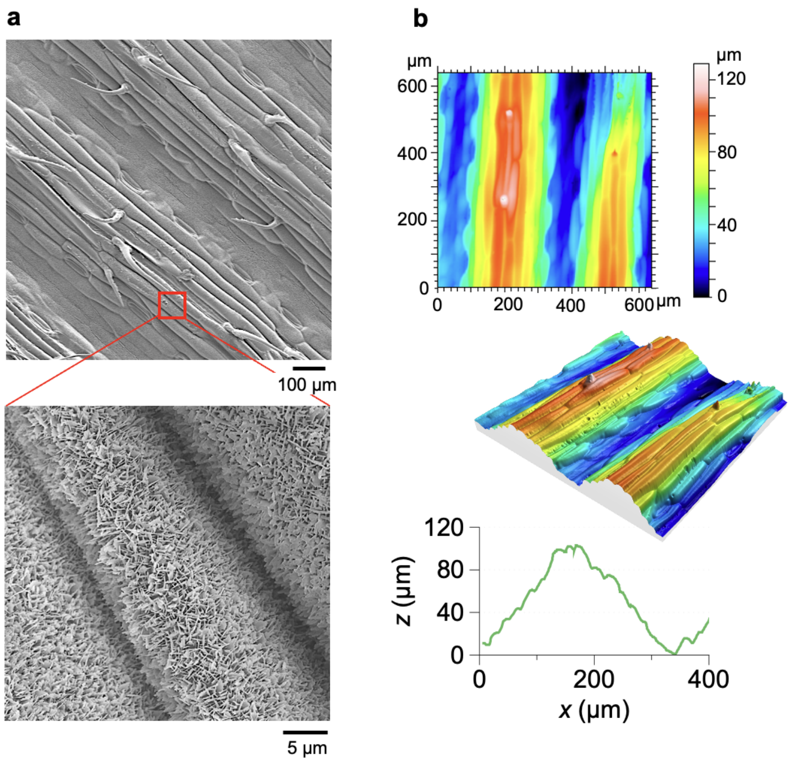

Figure S.1: Topography of wheatgrass leaf. (a) SEM images of the apex regions on the adaxial side of the leaves showing the top-view of undulations, trichomes and stomata on the leaf (top image). Bottom image shows a magnified top-view of the leaf populated with epicuticular wax crystals. (b) LSCM images showing the topographic view of a similar section of the leaf demonstrating the compound undulations.

2 Wax coverage and leaf age

The adaxial surface of the wheatgrass leaves was covered by a dense population of wax crystal platelets. No significant variation in the population, size, and shape of the crystals was found on the adaxial side of the fresh leaves after 5 days of growth, see Fig. S.2a, left. The wax crystal population was slightly reduced upon aging, probably due to mechanical abrasion or crystal coarsening, Fig. S.2a, right. For leaves of 5 days of age, no clear variation was observed in the wax coverage density for leaves of different heights in the population, see Fig. S.2b.

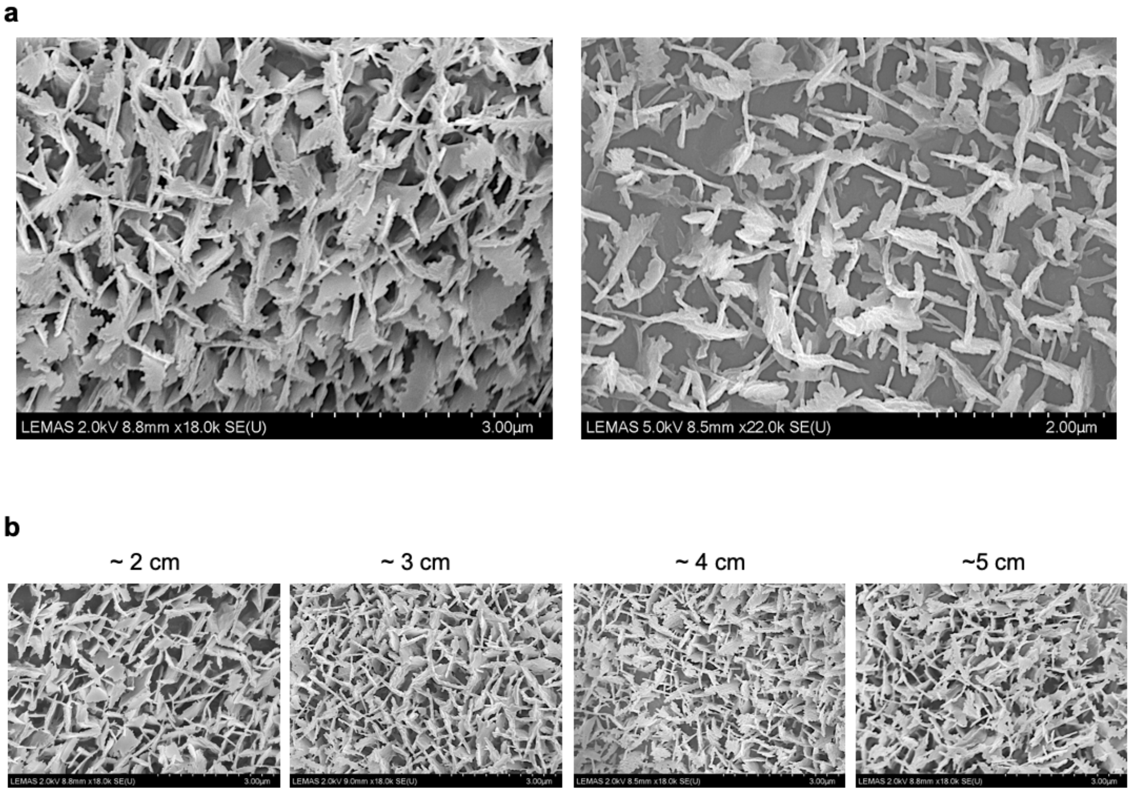

Figure S.2: SEM images of the apex region on the adaxial side of the wheatgrass leaves. (a) 7 days (left) and 16 days (right) old leaves. (b) Leaves of different height across a single population of 7 days old wheatgrass leaves.

3 Wax crystal structure

X-ray diffraction analysis was performed on the wax extracted from the wheatgrass leaves using chloroform. Data for pure 1-Octacosanol is provided for comparison. The diffraction intensity graph shows two intense short-spacing peaks highlighting the orthorhombic structure of the crystals, see Fig. S.3a. A bilayer structure is found in the long-spacing zone ($2\theta \leq 20°$), which has been attributed to the existence of the OH group of the primary alcohol that is the main component of the wheat leaf wax. Analysis of the diffraction intensity vector values of the long-spacing peaks shows a smaller slope for wheatgrass wax in comparison to 1-Octacosanol, Fig. S.3b. The long-chain bilayer spacing can be calculated using the slope of this graph via the relationship, $d_{\text{spacing}} = \frac{2\pi}{slope}$. The longer d_{spacing} obtained for wheatgrass wax may be due to the existence of longer chain compounds (primary alcohols and alkanes).

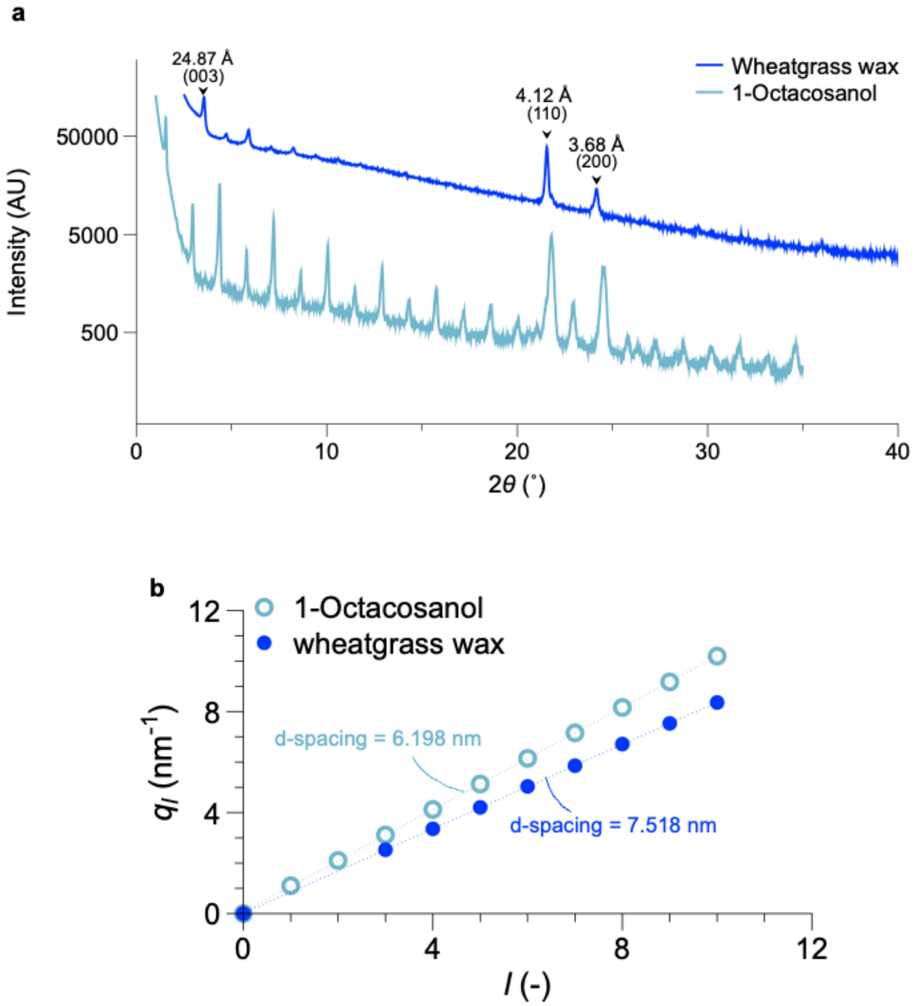

Figure S.3: (a) XRD results for the wax extracted from the wheatgrass leaf in comparison with pure 1-Octacosanol. (b) The magnitude of diffraction vector at the ordered long-spacing peaks q_L. The slope of the linear curve can be used to calculate the bi-layer spacing using $d_{\text{spacing}} = \frac{2\pi}{slope}$.

4 Role of trichomes

Although trichomes were found to be hydrophilic with little to no epicuticular wax, no initial nucleation was observed on their surfaces as cooling was provided from below (Fig. S.4a). As the droplets grow, the hydrophilic trichomes were found to trap the water condensation droplets (Fig. S.4b).

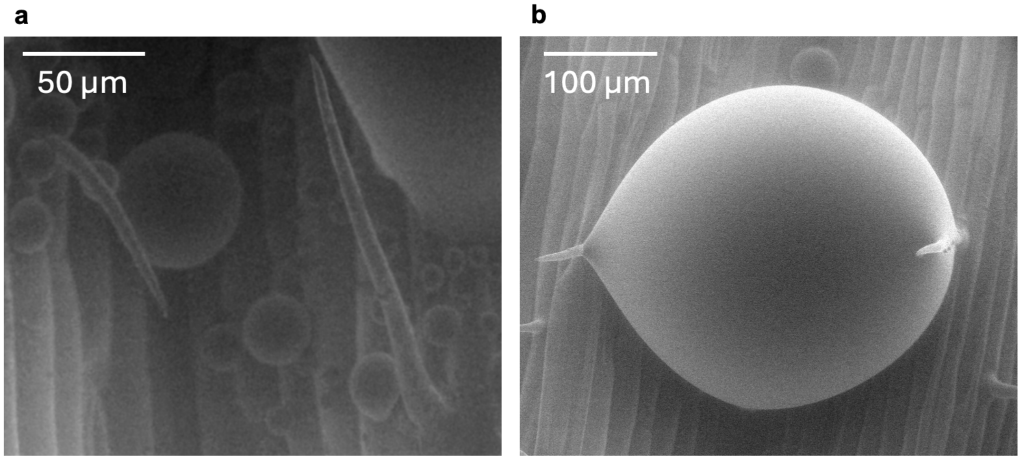

Figure S.4: Role of trichomes in condensation dynamics on the surface of wheatgrass leaves. (a) No initial condensation appears on the trichomes. (b) Trichomes were observed to trap larger condensation droplets.

5 Droplet growth dynamics

5.1 Spherical growth

During condensation spherical droplets formed on the adaxial surface of freshly harvested leaves (Fig. S.5a) and remained spherical throughout the growth period, see Fig. S.5b.

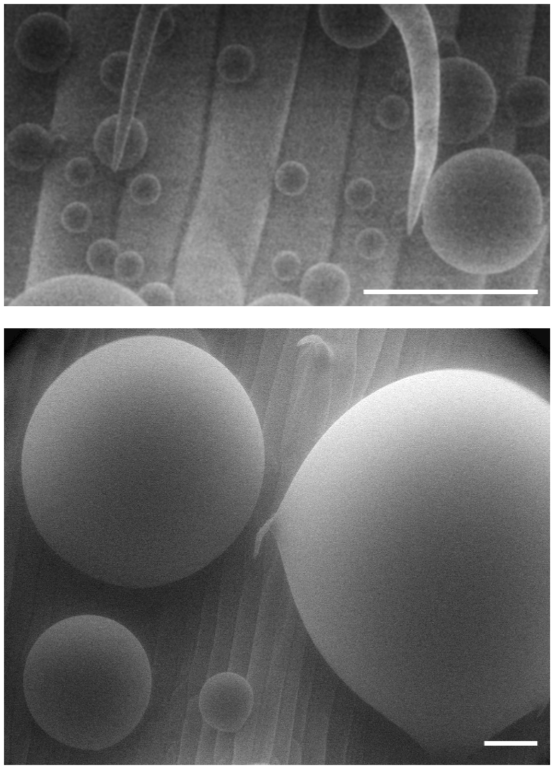

Figure S.5: ESEM images showing spherical growth of water condensation droplets on the adaxial surface of freshly harvested 7 days old wheatgrass leaves. Scale bars refer to 50 μm in both images.

5.2 Population density of droplet diameter

Droplet interface detection was performed on optical microscopy data. Temporal evolution of the population density of droplet diameter was used for tracking the regimes of growth during the subcooled condensation experiments (Fig. S.6a). At lower subcooling levels a single population with an increasing standard deviation shows growth through diffusion dominated process. Delayed nucleation events increase the polydispersity and standard deviation of the population at later times (Fig. S.6b).

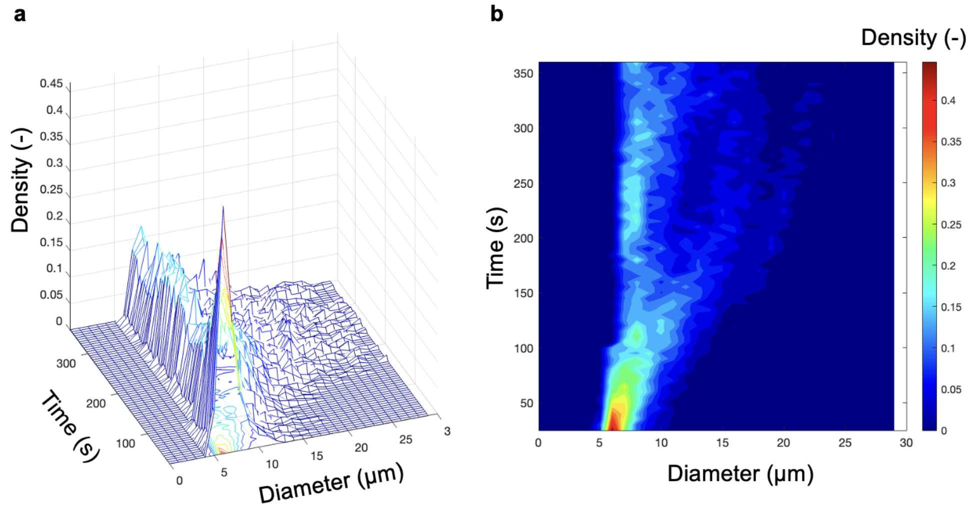

Figure S.6: Growth of condensation droplets on the adaxial surface of the leaf at ($\Delta T_c = 4$ °C). (a) Plot of the population density of condensation droplet diameter in time. (b) Contour plot representation of the same data.

5.3 Jumping droplets

Frequent droplet coalescence at lower subcooling temperatures leads to rapid ejection of the larger droplets. An example quantitative characterization of the radius of the droplets before and after the coalescence event for the jumping droplets discussed in Fig. 6c of the manuscript is presented in Fig. S.7. On average two to three initial droplets of 7.7 ± 1.3 μm radius were found to be involve in coalescence-induced jumping event, as illustrated by the number and magnintufe of the red bars in Fig. S.7. Droplet coalescence led to an average final radius of ≈ 10 μm for the jumping droplets presented by the blue bars in Fig. S.7.

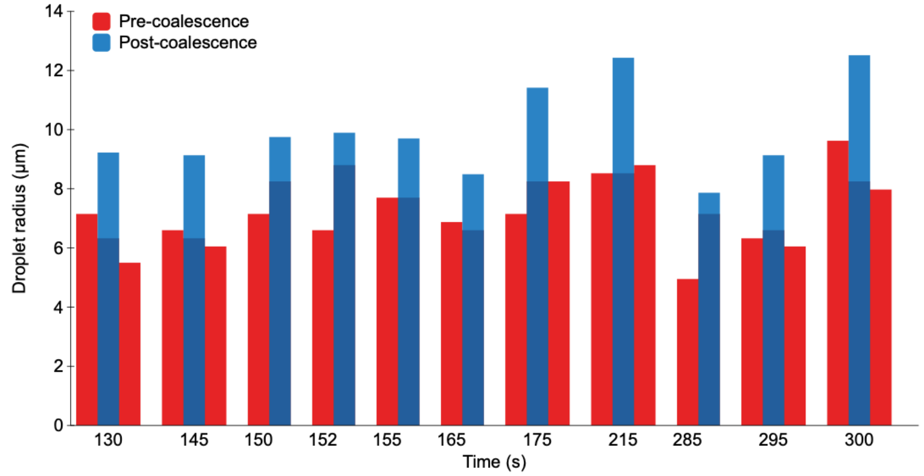

Figure S.7: Droplet radius before and after coalescence event quantified for jumping droplets observed at $\Delta T_c = 10$ °C. Analysis corresponds to the data presented in Fig. 6c of the manuscript.

5.4 Time-strip analysis

Time-strip analysis of optical microscopy images was performed after subtracting the initial background image. The grayscale intensities were analyzed and plotted along horizontal and vertical lines in the microscopy images, highlighting the growth of the bright droplet interfaces along the two axes. Fig. S.8 shows example results on the abaxial tip of the freshly harvested 7 days old leaves. At lower subcooling level, condensation droplets were observed to symmetrically grow throughout the experiments with rare coalescence events (Fig. S.8a). At higher subcooling level, relatively larger number of nucleation was observed yielding frequent coalescence events (Fig. S.8b).

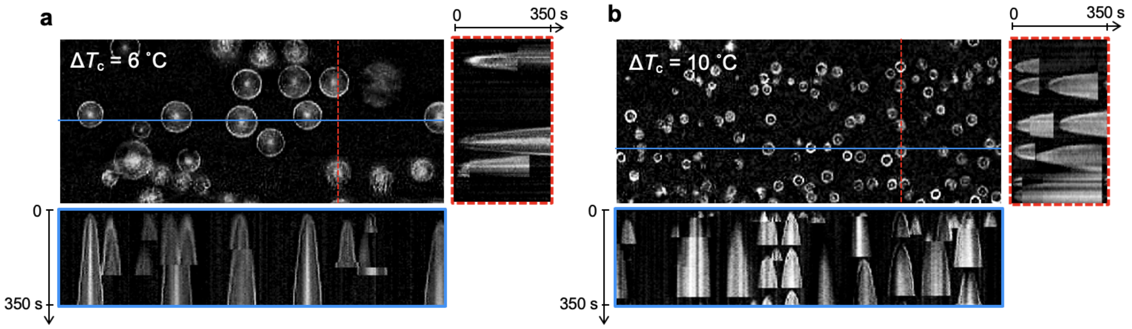

Figure S.8: Time-strip analysis of optical microscopy images on adaxial tip of freshly harvested 7 days old leaves at (a) $\Delta T_c = 6$ °C and (b) $\Delta T_c = 10$ °C.
